# Glycolytic Metabolism, Brain Resilience, and Alzheimer’s Disease

**DOI:** 10.3389/fnins.2021.662242

**Published:** 2021-04-28

**Authors:** Xin Zhang, Nadine Alshakhshir, Liqin Zhao

**Affiliations:** ^1^Department of Pharmacology and Toxicology, School of Pharmacy, University of Kansas, Lawrence, KS, United States; ^2^Neuroscience Graduate Program, University of Kansas, Lawrence, KS, United States

**Keywords:** Alzheimer’s disease, glycolysis, bioenergetics, biosynthesis, apolipoprotein E, diabetes, brain resilience

## Abstract

Alzheimer’s disease (AD) is the most common form of age-related dementia. Despite decades of research, the etiology and pathogenesis of AD are not well understood. Brain glucose hypometabolism has long been recognized as a prominent anomaly that occurs in the preclinical stage of AD. Recent studies suggest that glycolytic metabolism, the cytoplasmic pathway of the breakdown of glucose, may play a critical role in the development of AD. Glycolysis is essential for a variety of neural activities in the brain, including energy production, synaptic transmission, and redox homeostasis. Decreased glycolytic flux has been shown to correlate with the severity of amyloid and tau pathology in both preclinical and clinical AD patients. Moreover, increased glucose accumulation found in the brains of AD patients supports the hypothesis that glycolytic deficit may be a contributor to the development of this phenotype. Brain hyperglycemia also provides a plausible explanation for the well-documented link between AD and diabetes. Humans possess three primary variants of the apolipoprotein E (ApoE) gene – *ApoE^∗^ϵ2, ApoE^∗^ϵ3, and ApoE^∗^ϵ4* – that confer differential susceptibility to AD. Recent findings indicate that neuronal glycolysis is significantly affected by human ApoE isoforms and glycolytic robustness may serve as a major mechanism that renders an ApoE2-bearing brain more resistant against the neurodegenerative risks for AD. In addition to AD, glycolytic dysfunction has been observed in other neurodegenerative diseases, including Parkinson’s disease, Huntington’s disease, and amyotrophic lateral sclerosis, strengthening the concept of glycolytic dysfunction as a common pathway leading to neurodegeneration. Taken together, these advances highlight a promising translational opportunity that involves targeting glycolysis to bolster brain metabolic resilience and by such to alter the course of brain aging or disease development to prevent or reduce the risks for not only AD but also other neurodegenerative diseases.

## Alzheimer’s as a Metabolic Disease

The human brain contains an average of more than two hundred billion cells, one quadrillion connections, 100 km of nerve fibers, and 600 km of blood vessels (Steiner, 2019). Therefore, it is not surprising that a brain demands an outstanding amount of energy and building blocks to maintain its highly dynamic homeostasis. An adult brain makes up only 2% of total body weight, but it uses about 20% of total body energy (Costantini et al., 2008). The brain’s significant energy-consuming quality is largely attributed to the extremely active and complex processes involved in neuronal transmission. Failures to maintain basal energy levels, such as those under hypoglycemia or hypoxia, can potentially induce synaptic loss and cognitive impairment within a few minutes, thus rendering the brain exceedingly vulnerable to energy deficit-mediated damage (Fleck et al., 1993; Takata and Okada, 1995; Yamane et al., 2000).

Accumulating evidence indicates that in the development of AD, pathophysiological changes can occur up to 20–30 years before clinical symptoms manifest. Metabolic dysfunction has been recognized as a prominent anomaly in the brain during this preclinical stage (Small et al., 1995; de Leon et al., 2001; Mosconi et al., 2006, 2008b; Langbaum et al., 2009). The cerebral metabolic rate of glucose (CMRglc) is a critical indicator of neuronal and synaptic activity (Malonek and Grinvald, 1996; Attwell and Iadecola, 2002; Rocher et al., 2003; Khatri and Man, 2013). By means of positron emission tomography (PET) imaging and using 2-[18F]fluoro-2-deoxy-D-glucose (FDG) as the tracer, studies have shown that nearly all clinical AD symptoms are accompanied by significant reduction of CMRglc, and the extent and topography are correlated closely with symptom severity (Mosconi, 2005). Among individuals with mild cognitive impairment (MCI), the prodrome of AD, significantly decreased glucose metabolism has also been observed in AD-vulnerable brain regions, such as hippocampus, posterior cingulate cortex, and temporal cortex (Mosconi et al., 2008a,b). This condition reportedly predicts the progression from MCI to AD with greater than 80% accuracy. Additionally, individuals carrying an ApoE4 allele without dementia were found to exhibit a mild but definite reduction in CMRglc comparable to the typical AD pattern when compared to non-carriers (Small et al., 1995; Reiman et al., 1996, 2001, 2004; Perry et al., 2002; Mosconi et al., 2008a). This cerebral metabolic deficit substantially predisposes neurons to energy perturbation and functional crisis (Sims et al., 1980; Lying-Tunell et al., 1981; Hoyer et al., 1988; Filosto et al., 2007; Esteves et al., 2008; Vlassenko and Raichle, 2015; An et al., 2018; Vlassenko et al., 2018).

A great deal of research has sought to understand the biological basis responsible for the impaired glucose metabolism observed in the brains of AD patients and high-risk individuals. Glycolytic deficit has long been suggested as a prominent metabolic abnormality in the early stage of AD, evidenced by a much greater decline in cerebral glucose utilization when compared to the decrease in cerebral blood flow and the cerebral metabolic rate of oxygen (Hoyer et al., 1991; Fukuyama et al., 1994). These early findings have been solidified by recent studies, demonstrating that reduced glycolytic flux correlates closely with the severity of the disease, with more plaques and tangles found in the brains of AD patients (An et al., 2018). Moreover, studies have shown that a number of glycolytic elements, including glycolytic enzymes, glycolytic metabolites, and amino acids produced in the glycolytic pathway, are altered in AD. This suggests that glycolytic impairment associated with AD may cause both bioenergetic and biosynthetic disturbances that ultimately disrupt the metabolic and synaptic homeostasis, leading to abnormal protein deposition and cognitive decline (Sims et al., 1987; Fisher et al., 1991; Bigl et al., 1999; Hashimoto et al., 2004; Katsouri et al., 2011; Madeira et al., 2015; Wu et al., 2018). In this review, we summarize the current understanding of the function of glycolysis in the maintenance of brain health, and the role of glycolytic dysfunction as a possible cause of neurodegeneration in AD and other neurodegenerative conditions. We further discuss the research evidence that supports the emerging opportunity of targeting glycolysis as a potential therapeutic strategy aimed to bolster brain metabolic resilience and by such to alter the course of brain aging or disease development in the fight against the neurodegenerative risks for AD.

## Glycolysis Overview

### Bioenergetic Function of Glycolysis

**FIGURE 1:**
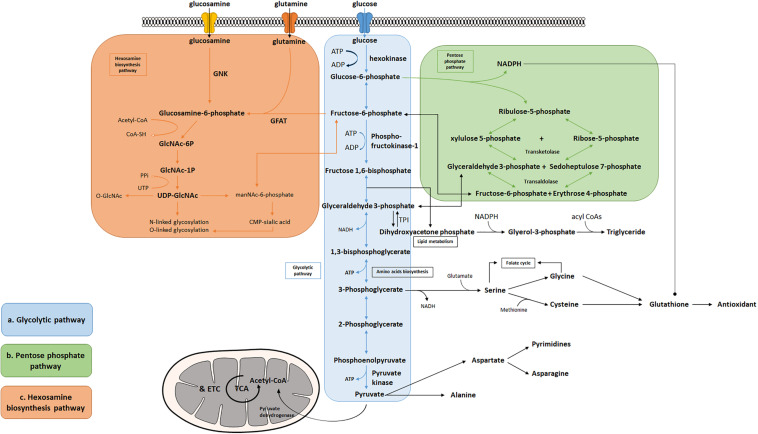
Bioenergetic and biosynthetic pathways of glycolysis. **(a)** Glycolysis breaks down glucose into two molecules of pyruvate accompanied by a net production of two molecules of ATP and 2 NADH + 2 H^+^. Metabolic intermediates derived from glycolysis serve as precursors in the biosynthesis of non-essential amino acids including serine, glycine, cysteine, and alanine. **(b)** The pentose phosphate pathway consists of two phases: oxidative and non-oxidative phases. Oxidation of G-6-P to pentose phosphates leads to the production of NADPH and five-carbon sugars, which are critical for reductive biosynthesis, antioxidant defense, and RNA/DNA synthesis. PPP is interconnected by transketolase and transaldolase with glycolysis. **(c)** Fructose-6-phosphate produced from G-6-P serves as the starting point that diverts 2–3% glucose to the hexosamine biosynthesis pathway, a branch derived from glycolysis, which generates the “sensing molecule” UDP-*N*-Acetylglucosamine (UDP-GlcNAc), the substrate of the enzymes involved in protein *N*- and *O*-glycosylation. The rate-limiting enzyme of the pathway is glutamine: fructose-6-phosphate amidotransferase (GFAT).

Many tissues can utilize fat or protein as a source of energy. Others, however, such as the brain, depend primarily on glucose to maintain normal functions (Maughan, 2009). Glycolysis is the cytosolic pathway in which one molecule of glucose is broken down into two molecules of pyruvate, along with a net production of two molecules of ATP and NADH (nicotinamide adenine dinucleotide). Pyruvates are then fully metabolized in mitochondrial respiration. The first five reactions of glycolysis constitute the preparatory or investment phase, where ATP is consumed, while the other five reactions form the payoff phase where ATP is produced (Figure 1a). Three key rate-limiting enzymes are utilized in glycolysis: hexokinase, phosphofructokinase-1 (PFK-1), and pyruvate kinase (PK). Each of them serves as a critical and tightly regulated site. In the initial step of glycolysis, hexokinase catalyzes the phosphorylation of glucose by ATP to produce glucose-6-phosphate (G-6-P), in which an ATP molecule is consumed. Hexokinase is feedback-inhibited by G-6-P as well as when the function of PFK-1 is suppressed, thus ensuring less hydrolysis of ATP and glycolytic intermediates. Notably, hexokinase possesses a low Km (high affinity, strong binding) for glucose which enables it to function actively even if the concentration of glucose is very low (Niemeyer et al., 1975; Massa et al., 2011).

Among the three key glycolytic enzymes, PFK-1 is regarded as the crucial point of regulation. PFK-1 converts fructose 6-phosphate to fructose 1,6-bisphosphate with consumption of another ATP molecule. This pathway is considered the commitment step to glycolysis and is allosterically regulated by the energy state of the cell. PFK-1 is inhibited by high levels of ATP and citrate, but activated when the ratio of ATP/AMP is low (Berg et al., 2007). When the body experiences acidosis (low pH), PFK-1 will also be suppressed to avoid excessive lactate production. In addition, fructose 2,6-bisphosphate acts as a potential activator for PFK-1 by enhancing its affinity for fructose 6-phosphate and inhibiting fructose-1,6-bisphosphatase, thus diminishing the inhibitory effect of ATP (Lunt and Vander Heiden, 2011).

The third, irreversible reaction is catalyzed by PK, which is also the final step of glycolysis, converting phosphoenolpyruvate to pyruvate and yielding two molecules of ATP. This ATP-forming reaction occurs by substrate-level phosphorylation and is highly regulated by the energy state of the cell. When the cell undergoes a high rate of glycolysis, fructose 1,6-bisphosphate can activate PK to keep up with the rate of glycolytic flux. Increased concentrations of downstream, energy-yielding intermediates, such as ATP or citrate, will inhibit PK and decelerate glycolysis (Berg et al., 2007). However, when cells are under energy deficit conditions, such as low blood glucose levels, phosphorylation of PK reduces its enzymatic activity, thereby limiting the consumption of glucose by the liver to meet the urgent energy demand of vital organs such as the brain (Pilkis et al., 1982).

Reducing equivalents, in the form of NADH, produced by glycolysis, are used to facilitate ATP production in the mitochondria by donating electrons to the electron transport chain. Cells normally rely on oxidative phosphorylation as the main source of energy (Zheng, 2012), however, the rate of ATP generation in the glycolytic pathway has been observed at a much higher level than oxidative phosphorylation in a number of different types of cells and tissues, such as muscle cells, neurons, astrocytes, microglia, endothelia cells, activated lymphocytes, or tumor cells (Pfeiffer et al., 2001; Pellerin et al., 2007; Pearce et al., 2013; Schmitz et al., 2013; Voloboueva et al., 2013; Ghesquiere et al., 2014). This phenomenon is essential to organisms when rapid ATP production is needed, such as muscle cells in heavy exercise or acute initiation of neuronal activities.

### Biosynthetic Function of Glycolysis

Producing energy is not the sole purpose of glycolysis. A wide variety of metabolic intermediates generated in the glycolytic pathway flow into a range of biosynthetic processes, including gluconeogenesis, lipid metabolism, the pentose phosphate pathway (PPP) and the TCA cycle. The Warburg effect has long been observed in rapidly proliferating cells and firstly described in cancer, where growing cells use glycolysis as the predominant pathway for ATP production even when oxygen is abundant (Warburg, 1956). Studies have demonstrated that induced chemotherapy-resistant cell lines, such as human LoVo colon carcinoma cells and HeLa, have elevated aerobic glycolysis (AG), indicating a mechanistic link between resistance and glycolysis (Ganapathy-Kanniappan and Geschwind, 2013). The Warburg effect may also play a role in anti-apoptotic effects in Aβ resistant neural cell lines via hypoxia inducible factor 1, indicating its protective role in AD brains (Newington et al., 2011). Thus, the significance of glycolysis extends beyond rapid energy generation both to facilitate nutrient assembly into essential precursors of biosynthesis and to promote cellular homeostasis.

Several amino acids are directly derived from glycolytic intermediates and play important roles in maintaining normal cellular function (Figure 1a). The carbon backbone that originates from 3-phosphoglycerate serves as the structural unit in the biosynthesis of serine, glycine, and cysteine, whereas pyruvic acid functions as the carbon provider for biosynthesis of alanine. Serine serves as one of the major sources for generation of nicotinamide adenine dinucleotide phosphate (NADPH) in the cell via the tetrahydrofolate (THF) cycle, supports cell proliferation via the regulation of pyruvate kinase, and functions as a head group when supplied directly to the biosynthesis of phosphatidylserine, a component of cell membrane in the brain (Lunt and Vander Heiden, 2011; Ye et al., 2012; Tedeschi et al., 2013; Fan et al., 2014; Lewis et al., 2014; Maddocks et al., 2014; Ducker et al., 2016; Gao et al., 2018). Moreover, the production of D-serine, a co-agonist of NMDA glutamate receptors, has been shown to be negatively controlled by glycolytic flux in astrocytes, via the interaction of serine racemase (an enzyme that converts L-serine to D-serine) and glyceraldehyde 3-phosphate, suggesting that glycolysis may play an important role in modulating excitatory neurotransmission in the brain (Suzuki et al., 2015; Guercio and Panizzutti, 2018). Le Douce et al. (2020) recently reported that glycolysis-derived L-serine production in astrocytes is impaired in AD. As the precursor of D-serine, reduced L-serine can cause D-serine deficiency leading to impaired NMDA receptor activity and synaptic and cognitive deficits (Le Douce et al., 2020). Alanine and aspartate play key roles in body function as well. Alanine transaminase (ALT) catalyzes the reversible reaction of glutamate and pyruvate into alanine and α-ketoglutarate, while aspartate transaminase catalyzes glutamate and oxaloacetate into aspartate and α-ketoglutarate. The subsequent alanine is then shuttled into the liver and enters the urea cycle. Aspartate can give rise to asparagine and the first committed step of pyrimidine biosynthesis by providing four atoms of the ring. The transamination reaction can also produce intermediatepyruvate and oxaloacetate, for gluconeogenesis thereby exerting its important role in both facilitating nutrient cycling and maintaining energy homeostasis.

A branch of glycolysis that accounts for 2–3% of total glucose metabolism is the hexosamine biosynthesis pathway (HBP) (Figure 1c) (Marshall et al., 1991). Fructose 6-phosphate, together with glutamine, is diverted to generate UDP-*N*-acetylglucosamine (UDP-GlcNAc). This end product of HBP is then used to form glycosaminoglycans, proteoglycans, and glycolipids (Schleicher and Weigert, 2000; Milewski et al., 2006; Yang and Qian, 2017). UDP-GlcNAc also functions as the substrate for O-linked *N*-acetylglucosamine transferases (OGTs) in various species involved in protein *N*- and *O*-glycosylation (Wells et al., 2001; Wells and Hart, 2003; McLarty et al., 2013; Hwang and Rhim, 2018). Glutamine: fructose-6-phosphate amidotransferase (GFAT) catalyzes the rate-limiting step in HBP conversion of fructose-6-phospate and glutamine, thus serving as an important regulatory point (Marshall et al., 1991). The regulation of GFAT, however, is not fully understood. A previous study suggests that glucose-6-phosphate dehydrogenase (G6PD) *O*-GlcNAcylation promotes the pentose phosphate pathway as well as cell proliferation and survival through an increased binding affinity of NADP^+^ to G6PD (Rao et al., 2015). *O*-GlcNAcylation is also reported to play a key role in regulating pyruvate kinase expression and activity leading to accumulation of upstream glycolytic metabolites (Chaiyawat et al., 2015; Wang et al., 2017). Overall, this evidence potentially suggests that glycolysis is an important mechanism underlying the regulation of the hexosamine biosynthetic pathway.

In addition to supporting UDP-GlcNAc biosynthesis, glycolysis also plays an important role in regulating triglyceride synthesis, by forming Glycerol-3-phosphate (G-3-P) through the reduction of dihydroxyacetone phosphate (DHAP) via G-3-P dehydrogenase (Zechner et al., 2012). G-3-P and fatty acyl CoAs are primary materials for *de novo* synthesis of glycerolipids, which are crucial for energy homeostasis, proper lipid transport, balancing glucose/lipid metabolism, and generation of metabolic signals (Prentki and Madiraju, 2008; Zechner et al., 2012). Research also finds that DHAP acts as an important precursor existing in the membranes of mitochondria and exhibits an important functional role in mitochondrial bioenergetics (Schlame et al., 1990, 1993; Paradies et al., 2014). Additionally, a deficiency of triose-phosphate isomerase (TPI or TIM), the enzyme catalyzing the rapid interconversion of DHAP and D-glyceraldehyde 3-phosphate (GAP), will lead to progressive neurological dysfunction and childhood mortality, thus highlighting its unique role in the process of glycolysis (Orosz et al., 2008).

## Glycolysis Functions in the Brain

The human brain depends mostly on glucose as the source of fuel, rendering it at great risk for neuronal dysfunction when glucose is in short supply. Apart from glucose, the brain can also utilize other energy substrates. Ketone bodies can provide energy (Owen, 2006; Zielke et al., 2009), but its fuel role is considered minor, except in times of starvation and glucose deprivation. Studies indicate that neurons predominantly undergo oxidative phosphorylation, whereas astrocytes are mainly responsible for lactate production via glycolysis (Hyder et al., 2006; Belanger et al., 2011). For the past 25 years, a prevalent viewpoint on neuroenergetics has been presented and developed: the astrocyte-neuron lactate shuttle (ANLS) (Pellerin and Magistretti, 1994). Upon intensified neuronal activity, glutamate is released into the synaptic cleft, followed by its action on postsynaptic receptors and uptake by astrocytes via excitatory amino acid transporters (EAAT). Glutamate in astrocytes is then converted by glutamine synthetase into glutamine, which is released by astrocytes, and taken up by neurons from the extracellular space. Within neurons, glutamine is converted to glutamate by glutaminase, packed in synaptic vesicles, and prepared to be released, thus the neurotransmitter pool of glutamate is replenished and the glutamate-glutamine cycle is completed (Mason, 2017). The uptake of glutamate by astrocytes is driven by a sodium gradient generated by Na^+^/K^+^ ATPase, of which ATP source is primarily from glycolysis (Pellerin and Magistretti, 1996). Theories suggest that neurons take up the extracellular lactate as the energy substrate from astrocytes in support of ATP generation upon the intensified neuronal activity (Magistretti and Allaman, 2018). The increased demand of energy accompanied by the glutamate-glutamine cycle induces higher glucose uptake from the circulation and enhanced lactate release by astrocytes, which attenuates extracellular prostaglandin E2 uptake, thus providing a potential mechanism underlying vasodilation and increased cerebral blood flow (Gordon et al., 2008; Howarth, 2014; MacVicar and Newman, 2015). A few studies have also indicated a neuroprotective role of an elevated brain lactate level, as evidenced by its ability in preventing neuronal excitotoxicity and decreasing lesion size in animal stroke models, possibly via an ATP- and redox-dependent pathway (Ros et al., 2001; Berthet et al., 2012; Jourdain et al., 2016; Margineanu et al., 2018). However, controversies exist showing neurons are capable of maintaining glutamatergic activity independent of the glutamate-glutamine cycle (Kam and Nicoll, 2007). Studies also showed that GABAergic neurons may not necessarily rely on lactate (Magistretti and Allaman, 2018). In addition, studies have also shown that, in response to increased neuronal activities, rather than obtaining lactate from astrocytes, neurons are capable of increasing glycolysis to produce lactate themselves, underscoring the role of glycolysis in sustaining neuronal function and maintaining homeostasis in the brain (Yellen, 2018).

### Glycolysis in Membrane Transport

It is a well-known fact that oxidative phosphorylation produces ATP in a much more efficient manner than does glycolysis. However, in acute neuronal events, glycolysis has been shown to become the dominant pathway for ATP generation (Fox et al., 1988). Proper functions of many ionic pumps such as Na^+^/K^+^-ATPase, H^+^-ATPase, and Ca^2+^-ATPase have been linked to membrane-bound glycolytic enzymes, both in the central nervous system and in other tissues, indicating that glycolysis-derived ATP may be an important source of energy for ion transport, which is critical for the conduct of action potential and synaptic transmission. V-type H^+^-ATPase (V-ATPase) is an ATP-dependent pump that mediates transmembrane transport of protons, thereby maintaining pH gradients between intracellular compartments, and V-ATPase is also required for proton secretion from the plasma membrane of certain specialized cells. V-ATPase has the highest expression in the brain and is a crucial constituent of synaptic vesicles. On the membrane of synaptic vesicles, V-ATPase pumps protons through the membrane into the synaptic vesicle, creating a proton concentration gradient, which is then used as an energy source, driving the movement of neurotransmitters into the vesicle through their respective transporters. Neurotransmitter concentration in the vesicle is an essential step preceding neurotransmitter release, underscoring the extremely essential role of V-ATPase in synaptic transmission (Moriyama et al., 1992). A number of studies have shown close interactions of V-ATPase with glycolytic enzymes, including PFK-1, Pfk2p, aldolase, and hexokinase, indicating the functional dependence of V-ATPase on glycolysis (Moriyama and Futai, 1990; Lu et al., 2001, 2004; Su et al., 2003; Nakamura, 2004; Kohio and Adamson, 2013; Chan et al., 2016; Woody et al., 2016).

Moreover, evidence exists that, following synaptic transmission, glutamate is quickly removed from the synaptic cleft by astrocytic uptake. This is an extremely efficient process in part powered by increased glycolysis that further increases the activity of Na^+^/K^+^-ATPase and the Na^+^-dependent cotransport uptake system in astrocytes (Pellerin and Magistretti, 1994). Furthermore, one study shows that when *Caenorhabditis elegans* neurons are under energy stress, a compensatory mechanism mediated by glycolysis maintains enough energy to support endocytosis and the synaptic vesicle cycle (Jang et al., 2016). Overall, despite the low yield of ATP produced by the glycolytic pathway, rapid ATP generation by glycolysis provides a significant advantage over oxidative phosphorylation, thereby playing a critical role for neuronal processes such as action potential, neurotransmitter release and uptake.

### Glycolysis in Postsynaptic Activity

A further role of glycolysis has been associated with postsynaptic density (PSD). PSD is a dense protein complex localized on the cell surface of post synapses in dendritic spines, containing receptors for almost all glutamate neurotransmission at excitatory synapses (Chen et al., 2015). The PSD clusters also regulate ion flux for Na^+^, K^+^, and Ca^2+^, adhesion proteins, scaffolding proteins, and protein kinases, including protein kinase A, protein kinase C, and Ca^2+^/CaM-activated protein kinase, along with their substrates. The rapid turnover and the dynamic property of PSD95 implies a great need for energy supply, as well as a significant quantity of intermediates for anabolism in dendritic spines, where, however, mitochondria are seldom present (Li et al., 2004). Instead, glycolytic enzymes are abundantly expressed in the PSD, including glyceraldehyde-3-phosphate dehydrogenase (GAPDH) and phosphoglycerate kinase (PGK), which may possibly be the source of ATP in the isolated PSD (Wu et al., 1997). Lactate is the end-product of anaerobic glycolysis functioning, and acts not only as a substrate for mitochondria, but also a second messenger to modulate the activity of neurons and astrocytes in neighboring regions. It is speculated that during acute neuronal activity, a significant interstitial lactate transient may be observed in areas where glutamate is released. However, when neuronal activity occurs, the diffusion and convection of lactate reaches much further than glutamate and the active zone, away from the area where glucose is predominantly consumed. Naïve neurons subsequently receive multiple signals, including lactate, which potentially augments inhibitory effects by adjacent GABAergic interneurons. This increased inhibition gives rise to limited glucose consumption in remote areas, thus recruiting more glucose to the active zone. The metabolic role of lactate in the brain is well discussed in a previous review (Barros, 2013). Monocarboxylate transporter 2 (MCT2), responsible for transporting lactate across the plasma membrane, has been reported to be present in PSD and co-localize with α-amino-3-hydroxy-5-methyl-4-isoxazolepropionic acid (AMPA) receptor GluR2/3 (Nusser et al., 1994; Bergersen et al., 2005; Goncalves et al., 2020). It is suggested that dysregulation of MCT causes axonal damage, amnesia, and memory deficits (Barros, 2013). These research findings provide strong support for the role of glycolysis in the regulation of postsynaptic activities.

### Glycolysis in Redox Homeostasis

Given that the brain is metabolically vulnerable, proper metabolic response of neurons is critical in defending the brain against injury, reactive oxygen species (ROS), and other neurodegenerative insults (Brand and Hermfisse, 1997; Rodriguez-Rodriguez et al., 2013; Butterfield and Halliwell, 2019). The pentose phosphate pathway (PPP) in glucose metabolism is a central source of pentoses and ribose 5-phosphate for cellular synthesis of nucleotides, as well as for reducing equivalents in the form of NADPH (Figure 1b). PPP accounts for about 60% of NADPH produced in a human body and is highly active in the liver, the adrenal cortex, and in red blood cells. NADPH is essential for cholesterol and steroid synthesis and respiratory bursts. In addition, NADPH is used by glutathione reductase to convert disulfide glutathione to reduced glutathione, which is in turn oxidized by glutathione peroxidase, coupled with the reduction of peroxides. Therefore, NADPH plays a critical role in maintaining the cellular redox state, which is tightly controlled by the rate-limiting enzyme of PPP, glucose-6- phosphate dehydrogenase (G6PDH). Coordination between glycolysis and PPP has been extensively described, including the rearrangement reactions in PPP that convert ribose-5-phosphate and xylulose-5-phosphate to fructose-6-P and glyceraldehyde-3-P under transketolase and transaldolase (Patra and Hay, 2014). Treatment with NADPH protects neurons against ROS and apoptosis, leading to increased ATP level, reduced long-term mortality, and improved functional recovery (Soucek et al., 2003; Mejías et al., 2006; Ying, 2008; Stanton, 2012; Li et al., 2016; Huang et al., 2018).

### Glycolysis in Brain Development

The PPP is a branch pathway from glycolysis after the first rate-limiting step catalyzed by hexokinase. Apart from its important role in antioxidant defense and nucleic acid synthesis, PPP also provides NADPH for reductive biosynthesis, such as biosynthesis of fatty acids and sterols (Figure 1b). Ranking as the second highest in lipid content after adipose tissue, human brain is particularly enriched with lipid content for maintenance of brain functions, such as synaptic activity, which in turn, renders it highly vulnerable to fatty acid and lipid disorders (Poitelon et al., 2020). In contrast to the peripheral system where lipids are the major form of energy storage, the lipids in the brain are primarily used for membrane construction, such as phospholipids (Hamilton et al., 2007). Synapse formation and elimination dynamically exist throughout a person’s entire life. A burst of synaptic formation occurs during early brain development, a phase known as exuberant synaptogenesis (Huttenlocher and Dabholkar, 1997). This synaptic dynamic is critical for normal synaptic connection and plasticity, proper neuronal network formation, task execution, learning process, and memory establishment. Moreover, studies show that during neuron differentiation, PFK-1 protein expression significantly increases, suggesting that glycolysis is necessary in supporting biosynthesis for neurite outgrowth and synaptic formation (Goyal et al., 2014; Agostini et al., 2016). In addition, a human brain represents only 2% of total body weight but contains 20% of the body’s cholesterol. Membrane lipids, primarily phospholipids, together with lipid composition of the myelin, comprise more than 50% of the brain solid matter, with concentration of brain phosphoglycerides being age dependent (Svennerholm, 1968). Dysregulation of lipid composition in the brain reportedly contributes to deterioration of CNS functions and pathological alteration in AD (Svennerholm, 1968; Söderberg et al., 1991; Velasco and Tan, 2014; Kao et al., 2020). Therefore, an impaired glycolytic pathway, such as dysregulated hexokinase, may reduce the essential intermediates and undermine PPP, thus inhibiting proper brain function and development.

The energy used by a developing brain is striking. Studies indicate the newborn’s brain is about 13% of body mass but consumes up to 60% of total body energy, and this soaring energy utilization lasts throughout one’s entire childhood. Notably, AG comprises 30% of glucose metabolism in a developing brain, compared to about 10% in an adult brain (Magistretti and Allaman, 2015), indicating an important role of glycolysis in brain development. Moreover, during pregnancy and infancy, brain volume and weight sharply increase, with brain size reaching about 75% of an adult’s brain by 2 years old, compared to 25% at birth (Steiner, 2019). Since neurogenesis mainly occurs prenatally—although some regions, such as the cerebellum, continue to generate after birth—rapid postnatal brain growth is mostly attributed to axon growth, dendritic morphogenesis, synaptic proliferation/elimination and axon myelination. This is a period when a brain meets both its highest energy demand and highest level of AG (Vlassenko and Raichle, 2015; Silbereis et al., 2016). Goyal et al. (2014) found that the elevated level of glycolysis during childhood correlates to the child’s highest rate of synaptic growth. They also discovered that in adult brain regions with the highest AG, genes that are responsible for synapse formation and growth are significantly increased (Vaishnavi et al., 2010; Goyal et al., 2014). Glycolysis is important in synaptic plasticity and as a link between glycolytic function and motor adaptive learning (Shannon et al., 2016). Other research shows that, in early postnatal mice, the neurite architecture was significantly impaired when glycolysis was pharmacologically inhibited (Segarra-Mondejar et al., 2018). Additionally, multiple researchers have demonstrated that glycolysis has a predominant role in elevated neuritic and synaptic formation, as well as their turnover, a role that presumably remains throughout human lifespan (Marder and Goaillard, 2006; Goyal and Raichle, 2013; Magistretti, 2014). Taken together, even though there are a limited number of studies, the developing brain is considered to be predominantly glycolytic. This is largely due to its dependence on *de novo* biosynthesis of lipids, amino and nucleic acids in support of developmental processes such as synaptogenesis, which ultimately leads to proper neuronal network that underlies cognitive function (Bauernfeind et al., 2014; Goyal et al., 2014; Steiner, 2019).

## Glycolysis in AD

Before reaching adulthood, brain glucose consumption is slightly reduced. Overall glycolysis, however, exhibits a much steeper decline from representing about 30% of glucose utilization to 8–10% (Vlassenko and Raichle, 2015; Steiner, 2019). Notably, AG appears to be spatially varied in the brain and is diminished topographically during normal aging. Regional variations are involved with reasoning, cognition, navigation, and executive motor control (Vaishnavi et al., 2010). Consequently, brain regions relying on high level of AG in young adults can be exceptionally vulnerable when approaching middle-age, given AG’s bioenergetic, biosynthetic, and neuroprotective role in the brain (Goyal et al., 2017).

### Correlation Between Aβ Deposition, Tauopathy, and Glycolysis

Accumulation of neurotoxic Aβ plaques and hyperphosphorylation of tau have long been considered the pathological hallmarks that contribute to synaptic disruption and neuronal loss in the brains of AD patients (Skovronsky et al., 2006; Querfurth and LaFerla, 2010; Serrano-Pozo et al., 2011; Krstic and Knuesel, 2013; Amtul, 2016; Harrison and Owen, 2016). It is reported that Aβ distributes variably among brain regions, with more deposition found in areas of high dependence on glycolysis. In a PET study of 33 neurologically healthy participants, Vaishnavi et al. (2010) discovered that AG was significantly elevated in the medial, lateral, and prefrontal cortices, whereas the cerebellum and medial temporal lobes exhibited lower glycolysis when compared to the mean value of the brain. Follow-up studies reported that the regions with increased glycolysis in the resting state of healthy young adults closely mirror the later regional pattern where Aβ accumulates in the brains of AD patients (Vaishnavi et al., 2010; Vlassenko et al., 2010; Goyal et al., 2020). The correlation observed in these studies is considered a compensatory mechanism in response to Aβ toxicity and mitochondrial dysfunction at a very early stage of AD. Another clinical study of 42 individuals aged 53–88 years at either preclinical or symptomatic stages of AD revealed close relationships among amyloid burden, AG, and tau deposition. Data showed that reduced synaptic plasticity and neuroprotection are related to the loss of AG, which may promote tauopathy in individuals with amyloid burden (Vlassenko et al., 2018). Studies using h-tau mice, which express all human tau isoforms, found that reduced glucose utilization, possibly via the downregulation of glycolysis, directly triggers tauopathy leading to synaptic dysfunction and behavior deficits (Lauretti et al., 2017). These results indicate the important role of brain glycolysis in the pathogenesis of AD.

### Altered Glycolytic Metabolite and *O*-GlcNAcylation in AD

A prospective ongoing cohort study that began in 1958 by the Baltimore Longitudinal Study of Aging (BLSA) investigated whether AD pathogenesis is correlated with dysfunction of glucose homeostasis. Glucose concentration and ratios of glycolytic amino acids (serine, glycine, and alanine) to glucose, which represent the cerebral glycolytic function, were measured within the autopsy cohort. The results showed that elevated brain glucose levels and reduced glycolytic flux are associated with the severity of AD pathology and expression of AD symptoms. They concluded that impaired glycolytic function may be intrinsic to glucose metabolic dysfunction inherent in AD pathogenesis (An et al., 2018). Another clinical study that analyzed 122 metabolites in the CSF of AD and non-AD subjects showed that only intermediates of glycolysis, such as dihydroxyacetone phosphate (DHAP) and phosphoenolpyruvate (PEP), were significantly decreased in AD patients. The reduction of these glycolytic intermediates also exhibited positive correlation with Aβ_1__–__42_ and Aβ_1__–__42_/Aβ_1__–__40_ (Bergau et al., 2019). A very recent study also reported that astrocytic glycolysis-derived L-serine exhibited a significant decrease in 3xTg-AD mice indicating a reduced glycolytic flux that led to impaired synaptic plasticity and memory (Le Douce et al., 2020).

A number of studies have looked at the roles of key glycolytic enzymes in glycolytic dysregulation of AD; the results, however, were mostly obtained from postmortem brain specimens of AD patients and are inconsistent. Hexokinase activity was reported to decrease significantly in brains, skin-cultured fibroblasts, and leukocytes of AD patients (Marcus et al., 1989; Sorbi et al., 1990). Whereas, in a paper of investigating hexokinase activity in a large Italian pedigree, the results showed no significant change of hexokinase activity (Mortilla and Sorbi, 1990). Studies of PFK showed that inhibition of fructose-2,6-biphosphatase (PFKFB3) led to Aβ accumulation in astrocytes and a higher risk of Aβ toxicity in cultures of human fetal astrocytes. In a study involving autopsy of AD patients, PFK activity was shown to decrease significantly to approximately 10% of the activity in the control group (Bowen et al., 1979). In another study, PFK activity in the frontal and temporal cortex of post-autopsy AD brains was found to be elevated when compared to age-matched non-AD individuals (Bigl et al., 1999). Furthermore, Sims et al. (1987) found no decrease of PFK activity in patients with primary degenerative dementia who are relatively young and at an early stage of the disease. The activity of the third key glycolytic enzyme, pyruvate kinase, is reported to significantly increase in the frontal and temporal cortex of AD brains (Bigl et al., 1999). However, other studies found a reduced level of pyruvate kinase activity in an age-dependent fashion in the frontal cortex of APP/PS1 mice (Harris et al., 2016). Factors that may have contributed to these inconsistencies include sample preparation procedure, age of the patients, AD study models, as well as the stage of AD.

Another key enzyme that has raised great interest in the past three decades regarding its role in neurodegenerative disease is GAPDH, a classic glycolytic enzyme that is primarily considered as the “housekeeping” protein and predominantly presents in the cytosol. GAPDH catalyzes the conversion of glyceraldehyde 3-phosphate to 1,3-bisphosphoglycerate in a NAD^+^-dependent manner and mediates the production of NADH + H^+^ and ATP. In a study measuring GAPDH activity in post-mortem brains of AD patients, the researchers discovered a ∼19% decline of GAPDH activity in temporal cortex (Kish et al., 1998). In line with the previous study, Mazzola and Sirover (2001) found a 27∼33% decline of GAPDH glycolytic activity in human skin fibroblast cell strains obtained from AD patients. However, controversies exist with studies showing that GAPDH enzymatic activity exhibited a significant increase in the frontal cortex lysate of six AD patients compared to an age-matched healthy group (Soucek et al., 2003). A possible explanation of the observed elevated GAPDH activity which contradicts the hypometabolism theory of AD brain could be the small sample size studied. Moreover, a human microglial cell line, CHME-5, showed an increased level of GAPDH upon the treatment of plasma from AD subjects (Jayasena et al., 2015). Increased glycolysis, however, is considered as a compensatory mechanism of impaired mitochondrial function following the exposure of AD plasma and functions as the forerunner of the energy deficit status in the cell. Furthermore, competing theories suggested that overexpression and aggregation of GAPDH serve as the pro-apoptotic regulator in neurodegenerative diseases, which possibly interacts with a voltage-dependent anion channel and mediating permeability transition of the mitochondria, a mechanism recognized as the non-glycolytic function of GAPDH (Ishitani and Chuang, 1996; Kish et al., 1998; Tarze et al., 2007; Nakajima et al., 2009; Itakura et al., 2015; Lazarev et al., 2020). Moreover, a heightened level of the reversible *S*-glutathionylation of GAPDH was found in the AD brain compared to age-matched control groups, which leads to the inactivation of GAPDH. The studies also showed the impaired enzyme activity can be rescued under certain reducing conditions, such as glutathione (Newman et al., 2007). Considering *S*-glutathionylation is an important post-translational modification in preventing protein from irreversible oxidation, a proposed theory points to GAPDH as an oxidative stress sensor, further implying a correlation between reduced glycolysis and the disturbed redox state of the cell (Gallogly and Mieyal, 2007). Additionally, inactivation of GAPDH has been considered to play a protective role under oxidative stress, by diverting carbohydrate flux to PPP, thus producing more reducing power, NADPH, against oxidants (Ralser et al., 2007). However, inactivated GAPDH also results in reduced energy. Therefore, in the long term when cells become exhausted by massive oxidative stress, a disastrous cascade effect that ultimately leads to cell death will be initiated by the cell’s inability to sustain energy demand and redox homeostasis. GAPDH was also found bonded on Inositol 1,4,5-trisphosphate receptors (IP3R) and proposed to mediate Ca^2 +^ release via NADH (Patterson et al., 2005). Hence, GAPDH also plays a significant role in regulation of Ca^2 +^ homeostasis, an important mechanism underlying AD pathogenesis, further linking perturbed glycolysis to cell death (Canzoniero and Snider, 2005; Bojarski et al., 2008).

Another area associated with glycolysis that has been examined in the context of AD is the HBP, also known as the nutrient sensing pathway (Zhu et al., 2014). Notably, O-linked *N*-acetylglucosamine (O-GlcNAc) is found greatly enriched in the brain, particularly at neuronal synapses, suggesting a role of the HBP in synaptic transmission (Zhang and Bennett, 1996; Vosseller et al., 2006; Skorobogatko et al., 2011; Trinidad et al., 2012). Moreover, increased *O*-GlcNAcylation has been demonstrated to promote neuroprotective outcomes such as reduced cerebral trauma, improved outcome of strokes, and alleviated stress (Groves et al., 2013; Gu et al., 2017). Using a human neuroblastoma cell model, upregulating *O*-GlcNAcylation led to an increased level of non-amyloidogenic sAPP α fragments and reduced Aβ secretion, suggesting *O*-GlcNAcylation of APP as an anti-Aβ target for AD (Jacobsen and Iverfeldt, 2011; Chun et al., 2017). *O*-GlcNAcylation has also been observed to inversely correlate with phosphorylation of tau, as demonstrated by fourfold less expression of *O*-GlcNAc in hyperphosphorylated tau than in non-hyperphosphorylated tau. Of particular note, rodent brains with impaired glucose metabolism exhibit changes in *O*-GlcNAcylation and tau phosphorylation that resemble those in the brains with inhibited HBP, suggesting the HBP is largely controlled by glucose metabolism (Liu et al., 2009). In sum, given that the HBP is a branch of glycolysis and that there is evidence that a decreased level of *O*-GlcNAcylation can result from impaired glucose metabolism, elevation of the HBP activity via boosting glycolysis may be a promising therapeutic approach in AD.

### Redox State, Glycolysis, and AD

Oxidative stress is a common feature in AD (Perry et al., 2002; Butterfield and Halliwell, 2019). NAD^+^ is a cofactor for redox reactions and plays an essential role in glycolysis to regulate cellular energy metabolism. Increasing evidence suggests that NAD^+^ is also involved in many other biological processes such as cell death, calcium homeostasis, gene expression, carcinogenesis, immunological functions, as well as aging (Ying, 2008). The NAD^+^/NADH ratio is an index of cellular reducing potential, dysregulation of which has been extensively indicated in AD. A DNA repair-deficient 3xTgAD/Polβ^±^ mouse model with exacerbated AD features, including tauopathy, synaptic dysfunction, neuronal death, and impaired cognitive function, exhibited a reduced cerebral NAD^+^/NADH ratio and indicated impaired energy metabolism. Treatment with nicotinamide riboside reversed the NAD^+^/NADH ratio and produced improved phenotypes, indicating that bolstering NAD^+^/NADH, potentially via glycolysis, may contribute to AD treatment (Hou et al., 2018). Another study using primary neuron culture showed that exposure to Aβ oligomers significantly reduced the NAD^+^ level and the decreased NAD^+^ level was rescued by nicotinamide treatment (Liu et al., 2013). Similar results were also observed in 3xTgAD mice (Liu et al., 2013). In a clinical study to evaluate the orally administrated NADH effect, 26 AD patients received NADH (10 mg/day) or a placebo. After 6 months of treatment, the NADH-treated group performed significantly better on the Mattis Dementia Rating Scale (MDRS) and the Mini Mental State Examination and showed no significant progression of AD symptoms, indicating the therapeutic value of NADH for AD (Demarin et al., 2004).

Moreover, the NADPH generated by PPP reacts with oxidized glutathione to form reduced glutathione (Figure 1b). The reduced glutathione further converts reactive H_2_O_2_ to H_2_O by glutathione peroxidase, thus maintaining cellular redox homeostasis. The role of PPP, a branched pathway directing from the first key step of glycolysis, in maintaining proper NADPH level and protecting against oxidative stress is thereby highlighted. In a study that involved 45 AD patients and 28 age-matched control subjects, both GSH levels and the GSH/GSSG ratio exhibited a profound reduction in the AD group (Bermejo et al., 2008). Additionally, Ivanov et al. (2014) reported that, during synaptic stimulation in hippocampal slices, a significant fraction of NAD(P)H response corresponded to glycolysis, suggesting that glucose serves as an effective energy substrate for both neurons and astrocytes in network activity. Considering the biosynthetic dependence of NADPH on glycolysis and its role in producing GSH from GSSG, proper function of glycolysis is vital not only for glucose metabolism, but also for maintaining redox homeostasis in AD.

### ApoE Isoforms Differentially Modulate Neuronal Glycolysis

As in many other chronic diseases, AD risk can be influenced by multiple factors, such as age, gender, family history, brain injury, environment, and lifestyle. Notably, results from postmortem autopsy showed that about 30% of cognitively normal people present various signs of AD pathology in the brain (Arenaza-Urquijo and Vemuri, 2018; Dumitrescu et al., 2020), raising the question of why some people have AD-like pathology but remain cognitively intact. Individual differences in overcoming adverse factors and thus maintaining a better performance can be a reason. This is referred to as brain resilience. Age-dependent reduced glycolysis in the brain occurs independent of amyloid plaques and serves as a biomarker for aging (Goyal et al., 2017). Given that aging is the greatest risk factor for the development of AD, enhancing glycolysis can potentially increase neuronal metabolic strength to sustain a better cognition and slow down or prevent AD progress. For instance, research has shown that neuronal cells with a higher level of glycolysis are more resistant to Aβ toxicity, indicating a neuroprotective effect mediated by glycolysis (Newington et al., 2011, 2012).

ApoE is the primary cholesterol carrier in the brain and must be produced locally. It is predominantly synthesized by astrocytes and to a lesser extent by microglia, vascular smooth muscle cells, and the choroid plexus (Uchihara et al., 1995; Achariyar et al., 2016). In addition, studies showed that under normal conditions, neurons also produce ApoE. This may be responsible for ∼20% of total ApoE protein levels in the cortex (Xu et al., 2006; Knoferle et al., 2014). Particularly, the intense induction of ApoE expression has been observed in injured or stressed neurons, indicating a critical role of this neuron-specific source of ApoE expression in cellular repair and maintenance (Boschert et al., 1999; Aoki et al., 2003). Human ApoE exists in three major alleles (ε2, ε3, and ε4) and each of them conveys different susceptibility for development of AD. ApoE2 is considered as neuroprotective, whereas ApoE4 is the greatest genetic risk for AD. Recent research led by Tarja Malm showed that human iPSC-derived microglia carrying ApoE4 exhibited a reduced extracellular acidification when compared to those carrying ApoE3, indicating an ApoE4-induced glycolytic deficit (Konttinen et al., 2019). Studies by Zhao et al. (2017) demonstrated that compared to ApoE3, ApoE4 secreted by astrocytes failed to induce insulin-stimulated glycolysis in *ApoE^–^/^–^* neurons. In clinical studies, ApoE4 has widely been associated with early onset of AD, rapid progression of the disease, more severe impairment of cognitive function and altered response to AD treatment (Morris et al., 1995; Nagy et al., 1995; Wilson et al., 2002; Wu and Zhao, 2016). Moreover, studies showed that ApoE2 carriers without dementia do not display the typical age-related increase of an Aβ burden (Grothe et al., 2017). Functional connectivity in the amygdala and entorhinal cortex tends to be increased and remain stable in individuals with ApoE2 allele (Gong et al., 2017). Attenuated hippocampal atrophy and a lower level of age-related myelin breakdown have also been observed in individuals carrying ApoE2, compared to non-carriers (Bartzokis et al., 2006; Chiang et al., 2010). The underlying mechanism of ApoE2 as a neuroprotective variant, however, remains largely unknown. Recent studies found that ApoE2-bearing mouse brains exhibit the most robust bioenergetic profile as evidenced by the highest levels of hexokinase expression and activity, glycolytic function, and ATP production when compared to both ApoE3 and ApoE4 mouse brains (Keeney et al., 2015; Woody et al., 2016; Wu and Zhao, 2016; Wu et al., 2018). In addition, ApoE2-mediated glycolytic robustness via the upregulation of hexokinase appears to directly correlate to a healthier cell status, which could serve as a major mechanism that allows ApoE2-bearing brains to be more resilient against AD (Zhang, 2018). In summary, growing evidence supports the idea that introduction of ApoE2 into the ApoE4 brain, such as by a gene or protein/peptide therapy, can be a plausible strategy to rescue the glycolytic deficits, improve cognitive function, and ameliorate AD-related neurodegeneration, thus helping close the therapeutic gap for AD patients.

## Brain Hyperglycemia as a Common Feature in Diabetes and AD

Diabetes has been well established as a risk factor for AD (Profenno et al., 2010). A great number of epidemiological studies revealed that being diabetic results in a higher risk of developing AD in later life, and such a risk is heightened when diabetes coexists with other risk factors such as the ApoE4 genotype. For example, in a Taiwanese population-based study of 615,532 diabetic patients and 614,871 non-diabetic individuals, the diabetic group showed a higher rate for developing AD over a period of 9 years with a hazard ratio of 1.45 relative to non-diabetics (Wang et al., 2012). In a longitudinal cohort study that followed up on 824 individuals older than 55 years, diabetes was associated with 65% higher incidence of AD over an average period of 5.5 years (Arvanitakis et al., 2004). Moreover, the Hisayama study followed up on 11,017 individuals older than 60 years for an average of 10.9 years and showed that impaired glucose tolerance and diabetes were respectively associated with 46 and 94% higher risk for developing AD (Ohara et al., 2011). In a community-based study whose subjects were obtained randomly from the Mayo Clinic Alzheimer Disease Patient Registry, the frequency of type 2 diabetes and impaired fasting glucose (IFG) was compared in 100 AD patients and 138 non-AD age-matched individuals. Results showed greater spread within the AD patients. Type 2 diabetes was present in 34.6% of AD patients vs. 18.1% of non-AD patients, whereas IFG was present in 46.2% of AD patients vs. 23.8% of non-AD patients (Janson et al., 2004). This indicates a possible common pathological link between the two diseases. Furthermore, the Honolulu-Asia aging study revealed that diabetes elevates the risk of developing AD in ApoE4 carriers compared to an isolated effect of ApoE4 without a diabetic condition (5.5 vs. 1.7 folds) (Peila et al., 2002). In light of the close association between AD and diabetes, the term “Type 3 Diabetes” has been used by some researchers as a label for AD (Steen et al., 2005; de la Monte and Wands, 2008). The underlying molecular mechanism, however, is not well understood. Research efforts directed toward better understanding of this relationship can ultimately pave the way toward better understanding of AD causative factors and thus to identification of key molecular targets that can be focused upon in future therapeutics.

Multiple studies have explored the relationship between blood plasma and brain glucose levels. An approach that has often been used is to alter plasma glucose levels within a predetermined range by dextrose injections, which is followed by measuring the levels of brain glucose that are achieved at different blood glucose levels. In a study performed on 18 healthy participants with mean age of 41 years, a linear relationship for glucose levels was shown between the cerebral and vascular compartments after stabilizing plasma glucose in a range of 4–30 mM: brain glucose levels proved to be 20–30% of plasma levels in the tested range (Gruetter et al., 1998). This plasma-to-brain glucose ratio and hyperglycemia-associated elevation of brain glucose are comparable to the data obtained in a study performed on white Wistar rats under normo- and hyper- glycemia (Silver and Erecinska, 1994). These findings clearly indicate that peripheral hyperglycemia induces a corresponding elevation of brain glucose levels. As hyperglycemia is one of the well-defined pathologies of diabetes, it has been hypothesized that the blood-brain barrier might adapt to chronic hyperglycemic states and thus limit the uptake of glucose into the brain as a protective mechanism. This hypothesis, however, was refuted in multiple studies which revealed that brain glucose levels are elevated in states of chronic diabetic hyperglycemia. For instance, in a study performed on 14 healthy individuals with mean age of 37, and 14 poorly controlled diabetic patients with mean age of 43 (type 1 = 8 patients, type 2 = 6 patients), hyperglycemia was induced in participants to an approximate blood glucose level of 300 mg/dL. This level produced brain glucose levels of 4.7 and 5.3 mM respectively in diabetics and non-diabetics (Seaquist et al., 2005). Another study performed on male Sprague Dawley rats showed similar results. Levels of glucose in brain extracellular fluid (ECF) under a hyperglycemic condition were compared to chronically hyperglycemic diabetic rats and non-diabetic rats. High plasma glucose (22 and 28 mM) led to comparable levels of brain ECF glucose in the two groups (mean brain ECF glucose: 7.5 and 8.7 mM respectively), while the mean of control-group normoglycemic rat brains ECF glucose was 2.1 mM at plasma glucose of 8 mM (Jacob et al., 2002). These data strongly indicate that diabetic hyperglycemia can directly result in brain hyperglycemia, regardless of the state of the disease.

As discussed earlier, a decreased glucose metabolic rate has been widely established as one of the main features of AD. However, the actual brain tissue glucose levels did not receive similar attention and the existence of a correlation between developing AD and alteration of brain tissue glucose levels was not explored until recent evidence from BLSA linked Alzheimer’s with accumulation of glucose in the brain (An et al., 2018). The study revealed that the brains of AD and asymptomatic AD patients, as opposed to brains of healthy participants, had higher levels of glucose—an alteration particularly prominent in brain regions of the cerebral cortex that are vulnerable to AD pathogenesis. Furthermore, brain tissue glucose concentrations were positively correlated with the severity of AD brain pathology. This and previous evidence lead to the conclusion that brain hyperglycemia is a shared feature of both diabetic and Alzheimer’s brains.

A considerable amount of evidence is available concerning the pathological impact of diabetes and hyperglycemia on the brain. Animal models of hyperglycemic diabetes have been shown to exhibit brain abnormalities that are comparable to dysfunction of Alzheimer’s brains, such as synaptic impairment (Malone et al., 2008; Liu et al., 2015), brain atrophy, and mitochondrial impairment (Carvalho et al., 2015). Furthermore, animal models having both diabetes and AD manifest an exacerbated level of brain pathology and cognitive impairment compared to models having AD only. A study that compared the effects of diabetes versus AD on the brain showed similar phenotypes in the brains of both a sucrose-induced mouse model of diabetes and a triple transgenic AD mouse model (3xTg-AD), including reduced brain weight, mitochondrial dysfunction, and reduced levels of synaptic and autophagy-related proteins when compared to WT animals (Carvalho et al., 2015). Activity of Na+/K+ ATPase, an essential pump that maintains neuronal resting membrane potential and its activity, was shown to be impaired by hyperglycemia in isolated synaptic terminals of aged Wistar rats’ brains (Torlinska et al., 2006). In a streptozotocin-induced hyperglycemic rat model of diabetes, neuronal spine density and dendritic branches were reduced compared to WT rats. The reduction was accompanied by memory impairment in the Morris water maze cognitive test (Malone et al., 2008). Synaptic plasticity as measured by long-term potentiation (LTP) was decreased in a high-fat diet-induced hyperglycemic mouse model (Liu et al., 2015). Animals with both diabetes and AD attained worse outcomes in the Morris water maze test, LTP, and mitochondrial respiration and enzymatic activities of complexes I and IV, when compared to animals with only diabetes or AD (Wang et al., 2015). In a similar study, comorbidities of diabetes and AD led to increased formation of Aβ oligomers and deposition of Aβ plaques, increased tau pathology, exacerbated neuroinflammation, and worsened performance on the Morris water maze test when compared to animals having only diabetes or AD (Guo et al., 2016). Overall, considering the hyperglycemic phenotype and other associated pathological features shared in brains of animals having both diabetes and AD, it is probable that brain hyperglycemia serves as a mechanistic link between the two diseases and contributes to AD development. Glycolytic deficit could be a major cause of increased glucose accumulation and ultimately hyperglycemia in non-diabetic AD brains.

## Glycolytic Dysfunction in Other Neurodegenerative Diseases

Glycolytic dysfunction has been associated with other neurodegenerative diseases, including Parkinson’s disease (PD), Huntington’s disease (HD), and amyotrophic lateral sclerosis (ALS). In the glycolytic pathway, phosphoglycerate kinase (PGK) is a major enzyme that catalyzes the first ATP-generating step in which a phosphate group in 1,3-biphosphoglycerate is transferred to ADP, producing 3-phosphoglycerate and one molecule of ATP. Deficiency of PGK activity caused by genetic mutations (e.g., c.649G > A) that results in impaired ATP production had been established as a major cause of medical conditions such as hemolytic anemia, myopathy, and neurological deficits (Matsumaru et al., 2017). Multiple studies have reported that patients suffering from these disorders exhibited PD-like symptoms, pointing to the role of PGK deficiency in the development of idiopathic PD (Sotiriou et al., 2010; Sakaue et al., 2017; Cai et al., 2019; Le Bras, 2019; Shimizu et al., 2020). For example, in one case report, a child with PGK1 deficiency developed parkinsonism at 9 years of age, whereas the mother, a heterozygous carrier of the mutation, developed parkinsonism at 36 years of age, suggesting a gene dose-dependent effect of PGK1 deficiency in conferring susceptibility to PD (Sakaue et al., 2017). Similarly, a 25-year-old male carrier of a PGK1 mutation that caused a marked decrease in PGK activity presented both exertional myoglobinuria and severe parkinsonism that was responsive to levodopa treatment (Sotiriou et al., 2010). These clinical findings have been further validated in molecular studies conducted in preclinical models. In a *Drosophila* model, dopaminergic (DA) neuron-specific PGK knockdown led to locomotive defects accompanied by significant reductions in ATP and dopamine levels, and progressive loss of DA neurons (Shimizu et al., 2020). Furthermore, in a variety of either toxin-induced or genetic PD models as well as in iPSC, treatment with terazosin, a PGK agonist, increased brain ATP and dopamine levels and restored motor function, providing support for the therapeutic approach of enhancing PGK and glycolytic activity in the treatment of PD (Cai et al., 2019).

Altered glycolytic metabolism has been observed in HD as well, although discrepancies exist. In an iPSC-based model of the disease, it was found that when compared to control cells, HD cells had decreased ATP levels, lowered expression of glycolytic enzymes, and decreased spare glycolytic capacity. In contrast, both mitochondrial messenger levels and protein levels, as well as respiratory capacities driven by oxidative phosphorylation, were largely unchanged. Moreover, ATP levels in HD cells were restored by treatment with pyruvate or late glycolytic intermediates, but not earlier glycolytic metabolites, providing further evidence for glycolytic and not mitochondrial deficits associated with HD (The HD iPSC Consortium, 2019). In agreement with these studies, Powers et al. reported a significant increase in the molar ratio of cerebral oxygen metabolism to cerebral glucose metabolism [CMRO(2)/CMRglc] in the striatum of HD patients, and the group postulated that glycolytic reduction in striatal metabolism could be involved in the pathogenies of HD (Powers et al., 2007). On the contrary, in HEK293 cell lines and transgenic *Drosophila* expressing polyglutamine (polyQ) in exon 1 of the huntingtin (HTT) protein, Sameni et al. (2016) observed an increased glycolytic rate, as indicated by an increased production of free NADH, in cells and tissues that expressed the expanded HTT-polyQ, when compared to controls that expressed unexpanding HTT-polyQ. In another study, it was found that the WNT/β-catenin pathway was increased in both HD and ALS, which increased activation of several glycolytic enzymes, which in turn resulted in increased glycolysis (Alexandre et al., 2018). An independent investigation conducted by Manzo et al. (2019) in a *Drosophila* model of TDP-43 proteinopathy demonstrated that increased glycolysis may serve as a compensatory mechanism that neurons attempt to use to fight against metabolic deficits in ALS. Further studies are certainly required to resolve these inconsistencies. Nevertheless, these findings clearly indicate a prominent involvement of glycolytic metabolism in the development of these neurodegenerative conditions.

## Conclusion

Alzheimer’s disease has long been recognized as a metabolic disease (Drzezga et al., 2003; Chen and Zhong, 2013; de la Monte and Tong, 2014). In particular, glucose hypometabolism has been established as a prominent anomaly in the very early development of the disease (Mosconi et al., 2008c). As the primary substrate of energy in the brain, glucose is first metabolized in the cytoplasmic glycolytic pathway, followed by oxidative phosphorylation in the mitochondria. Beyond its bioenergetic function, glycolysis plays crucial roles in many biosynthetic processes, for example, in the production of certain amino acids, ribose phosphate, and reduced glutathione, as well as in the glycosylation modification of proteins and lipids. Specifically, glycolysis has been extensively described for its essential roles in brain development and fast-occurring neuronal activities such as ion transport in neurotransmission. Despite its essential neural functions, glycolysis in the context of AD has not been explored much until recently (Bergau et al., 2019; Butterfield and Halliwell, 2019; Theurey et al., 2019; Yan et al., 2020). Several clinical studies have indicated the involvement of glycolytic dysfunction in the development of AD pathologies (Vlassenko et al., 2018). Moreover, increased brain glucose accumulation has been validated in AD patients, supporting the hypothesis that glycolytic deficit as an important contributor to the development of this phenotype (An et al., 2018). Brain hyperglycemia also provides a plausible explanation for the well-documented link between AD and diabetes. Human ApoE exists as three isoforms, ApoE2, ApoE3, and ApoE4. Carrying ApoE4 is the greatest genetic risk factor for sporadic AD, whereas ApoE2 carriers are resistant to AD. Historically, extensive research has focused on the neurotoxic effect of ApoE4, leaving ApoE2 largely unexplored (Wu and Zhao, 2016). Recent studies have provided several lines of evidence supporting the hypothesis that differential regulation of neuronal glycolysis could serve as one significant mechanism that underlies the different AD risk of ApoE isoforms (Wu et al., 2018). Glycolytic robustness, in large part via upregulation of hexokinase, could play a critical role in conferring ApoE2-bearing brains their resilience to AD. Besides AD, glycolytic dysfunction has been observed in other neurodegenerative diseases, including PD, HD, and ALS, strengthening the concept of glycolytic dysfunction as a common pathway leading to neurodegeneration. Taken together, these advances highlight an exciting translational opportunity not only for the AD field but also for research fields of other neurodegenerative diseases.

## Author Contributions

All authors listed have made a substantial, direct and intellectual contribution to the work, and approved it for publication.

## Conflict of Interest

The authors declare that the research was conducted in the absence of any commercial or financial relationships that could be construed as a potential conflict of interest.

## Glossary

AD, Alzheimer’s disease; ApoE, apolipoprotein E; CMRglc, cerebral metabolic rate of glucose; PET, positron emission tomography; FDG, 2-[18F]fluoro-2-deoxy-D-glucose; MCI, mild cognitive impairment; ATP, adenosine triphosphate; NAD, nicotinamide adenine dinucleotide; PFK-1, phosphofructokinase-1; PK, pyruvate kinase; G-6-P, glucose-6-phosphate; AMP, adenosine monophosphate; PPP, pentose phosphate pathway; TCA cycle, tricarboxylic acid cycle; AG, aerobic glycolysis; Aβ, amyloid beta; NADP, nicotinamide adenine dinucleotide phosphate; THF, tetrahydrofolate; NMDA, *N*-methyl-D-aspartate; ALT, alanine transaminase; HBP, hexosamine biosynthesis pathway; UDP-GlcNAc, UDP-*N*-acetylglucosamine; OGTs, O-linked *N*-acetylglucosamine transferases; GFAT, glutamine: fructose-6-phosphate amidotransferase; G6PD, glucose-6-phosphate dehydrogenase; G-3-P, glycerol-3-phosphate; DHAP, dihydroxyacetone phosphate; TPI or TIM, triose-phosphate isomerase; ANLS, astrocyte-neuron lactate shuttle; EAAT, excitatory amino acid transporters; V-ATPase, V-type H^+^-ATPase; Pfk2p, phosphofructokinase-2 subunit β; PSD, postsynaptic density; GAPDH, glyceraldehyde-3-phosphate dehydrogenase; PGK, phosphoglycerate kinase; MCT2, monocarboxylate transporter 2; AMPA, α-amino-3-hydroxy-5-methyl-4-isoxazolepropionic acid; ROS, reactive oxygen species; G6PDH, glucose-6- phosphate dehydrogenase; BLSA, baltimore longitudinal study of aging; CSF, cerebrospinal fluid; PFKFB3, fructose-2,6-biphosphatase; IP3R, inositol 1,4,5-trisphosphate receptors; O-GlcNAc, O-linked *N*-acetylglucosamine; GSH/GSSG, reduced glutathione/oxidized glutathione; iPSC, induced pluripotent stem cell; IFG, impaired fasting glucose; ECF, extracellular fluid; WT, wild type; LTP, long-term potentiation; PD, Parkinson’s disease; HD, Huntington’s disease; ALS, amyotrophic lateral sclerosis; DA, dopaminergic; CMRO(2)/CMRglc, ratio of cerebral oxygen metabolism to cerebral glucose metabolism; polyQ, polyglutamine; HTT, huntingtin.
